# Competing risk of death in patients with low, intermediate and high risk of recurrence after radical surgery for clear cell renal cell carcinoma

**DOI:** 10.1002/bco2.70047

**Published:** 2025-07-21

**Authors:** Anna Brännbäck, Ivan Mustonen, Teemu D. Laajala, Paula Vainio, Magnus Lindskog, Anders Kjellman, Per‐Olof Lundgren, Panu M. Jaakkola, Kalle E. Mattila

**Affiliations:** ^1^ Department of Clinical Science, Intervention and Technology, Karolinska Institute Karolinska University Hospital Stockholm Sweden; ^2^ Department of Oncology and Radiotherapy and Fican West Cancer Centre University of Turku and Turku University Hospital Turku Finland; ^3^ Department of Mathematics and Statistics University of Turku Turku Finland; ^4^ Research Program in Systems Oncology University of Helsinki Helsinki Finland; ^5^ Department of Pathology University of Turku and Turku University Hospital Turku Finland; ^6^ Department of Pelvic Cancer, Genitourinary Oncology Karolinska University Hospital Stockholm Sweden; ^7^ Department of Oncology‐Pathology Karolinska Institutet Stockholm Sweden; ^8^ Department of Immunology, Genetics and Pathology Uppsala University Uppsala Sweden; ^9^ InFLAMES Research Flagship Center University of Turku Turku Finland

**Keywords:** adjuvant therapy, kidney cancer, mortality, nephrectomy, prognostic models, risk factors

## Abstract

**Objectives:**

Adjuvant pembrolizumab has improved overall survival after surgery for clear cell renal cell carcinoma (ccRCC) with an intermediate‐high and high risk of recurrence according to the inclusion criteria of Keynote‐564 study, but non‐RCC mortality is common during postoperative follow‐up. We aimed to evaluate the competing risk of death after surgery in patients with ccRCC stratified according to the risk of recurrence with Keynote‐564, Three‐feature and Leibovich models.

**Material and Methods:**

A total of 1108 patients with ccRCC operated with curative intent between 2005 and 2021 before the use of adjuvant immunotherapy were identified from two academic centres in Finland and Sweden. Patients with cytoreductive nephrectomy, multiple kidney tumours or non‐ccRCC were excluded. Baseline characteristics and survival outcomes were described, and the Kaplan–Meier method was used to estimate overall survival.

**Results:**

During the median postoperative follow‐up of 5.0 years, 134 (12%) patients had died from RCC with a median time to death of 3.7 years (IQR 1.6–6.6) while for 220 (20%) patients the cause of death was other than RCC, most commonly other cancers (n = 59, 5%) and cardiovascular diseases (n = 54, 5%). According to the Keynote‐564 criteria, 34 (3%) patients were classified as having high risk of recurrence, 336 (30%) patients intermediate‐high risk and 738 (67%) patients low risk of recurrence with 41% of RCC deaths observed in this subgroup. Limitations of this study include the lack of information on performance status, comorbidities and systemic treatments for recurrent RCC.

**Conclusions:**

In addition to deaths from RCC, deaths from other cancers and cardiovascular diseases were common after surgery for ccRCC. As 41% of RCC deaths were observed among patients currently excluded from adjuvant therapy, more research on patient selection for perioperative immunotherapy is needed as well as interventions improving the treatment of comorbidities and lifestyle after nephrectomy.

## 1 INTRODUCTION

Every year, over 400 000 new cases of renal cell carcinoma (RCC) are diagnosed leading to over 175 000 deaths globally.^1^ The highest incidence of RCC is found in North America and in northern Europe.^2^ Clear cell RCC accounts for 75–80% of cases and is associated with inferior survival compared to papillary and chromophobe subtypes.^1^, ^3^ Localized RCC is treated with radical or partial nephrectomy (RN, PN) with curative intent. However, 11–30% of patients present with synchronous metastases and as much as 40% will develop recurrence after surgery and are at risk of death from metastatic RCC (mRCC).^4^, ^5^ Larger tumour size, higher grade, tumour necrosis, sarcomatoid differentiation, vascular invasion and lymph node involvement are associated with a greater risk of metastases,^6^, ^7^, ^8^ and these variables are utilized in prognostic models aiming to guide postoperative follow‐up and patient selection for adjuvant therapy.^9^

Since the 1990s, there has been a shift towards earlier detection of kidney tumours. Patients diagnosed with stage I and II RCC (60–70% of all cases) have five‐year survival rates of over 80%.^4^, ^8^, ^10^ Patients with stage III or IV have poorer prognosis, and the five‐year survival rate remains at <30% in stage IV RCC even after the introduction of angiogenic receptor tyrosine kinase inhibitors (TKI).^10^ However, immune checkpoint inhibitors (ICI) and ICI − TKI combinations have prolonged overall survival (OS) in mRCC,^11^ and adjuvant pembrolizumab has improved OS in patients with intermediate‐high or high risk of recurrence after surgery for ccRCC.^12^ Thus, patients are expected to live longer after RCC diagnosis.

As the incidence of RCC peaks between 60 and 75 years and male sex, smoking, obesity, hypertension and chronic kidney disease are also associated with RCC,^1^, ^2^, ^13^ patients may harbour comorbidities and risk factors predisposing to death from other causes. In the US, it was found that 51% of deaths of RCC patients were due to RCC, 11% died from other cancers and 38% of deaths were due to non‐cancer causes.^14^ 86% of deaths were attributed to RCC in patients with stage IV or metastatic disease, whereas 61–66% of patients with stage I or localized RCC died from non‐cancer causes, most commonly cardiovascular diseases (CVD).^14^ Among elderly Finnish patients with RCC, 55% died from RCC and 23% died from other causes during the median follow‐up of two years.^15^

To evaluate the accuracy of different prognostic models in predicting RCC mortality, we determined competing risk of death for two independent patient cohorts surgically treated with curative intent for clear cell RCC before the era of adjuvant immunotherapy at two academic centres in Finland and in Sweden. Patients were stratified into risk groups for disease recurrence as defined in the Keynote‐564 study, Three‐feature and Leibovich models.^7^, ^12^, ^16^

## 2 MATERIAL AND METHODS

### 2.1 Inclusion criteria

Two cohorts of patients were defined from electronic medical records with the same inclusion criteria. Study patients had undergone surgery with curative intent for ccRCC (M0 or complete resection of metastases at the time of nephrectomy or within one year after nephrectomy [M1 NED]). Patients with cytoreductive nephrectomy and multiple kidney tumours were excluded. Non‐clear cell RCCs were excluded because of different prognostic characteristics.^5^, ^6^

### 2.2 Turku cohort

Surgical and oncological treatment of RCC and postoperative follow‐up is centralized at Turku University Hospital in Southwest Finland. All patients with RCC (ICD‐10 code C64.88) who had undergone surgery from January 2005 to December 2021 (n = 593) were identified. After excluding 59 patients with non‐ccRCC, 54 with cytoreductive nephrectomy and 7 with multiple kidney tumours, the final Turku cohort consisted of 473 patients. The follow‐up period ended in December 2023.

### 2.3 Stockholm cohort

Karolinska University Hospital is a tertiary referral hospital and the largest centre treating RCC in Sweden. All patients treated for RCC between 2005 and 2023 were screened from the institutional database. Altogether 2303 patients with RCC were identified. After excluding 875 patients with non‐ccRCC, 141 were treated with tumour ablation, 502 with M1 RCC treated without curative intent, 9 with multiple kidney tumours, 17 with surgical treatment for cancer recurrence, 18 with missing data and 106 with less than two years of follow‐up, 635 patients remained in the final Stockholm cohort. The follow‐up period ended in April 2024.

### 2.4 Clinical characteristics

Baseline characteristics including age, sex, surgical procedure (RN/PN) and date of surgery were collected from electronic medical records. Tumour size, pT stage, pN stage, M stage (M0, M1 NED), Fuhrman and WHO/ISUP grades, macrovascular invasion into the renal vein and vena cava, microvascular invasion defined as tumour cells within small vessels in tumour pseudocapsule, tumour or renal parenchyma adjacent to the tumour,^17^ histologic tumour necrosis and sarcomatoid differentiation were obtained from pathology reports. Postoperative follow‐up was performed according to the local clinical practice. Information on RCC recurrence (date and site) and survival (date and cause of death or the last visit if alive at the end of the follow‐up) were obtained.

### 2.5 Statistical methods

**FIGURE 1:**
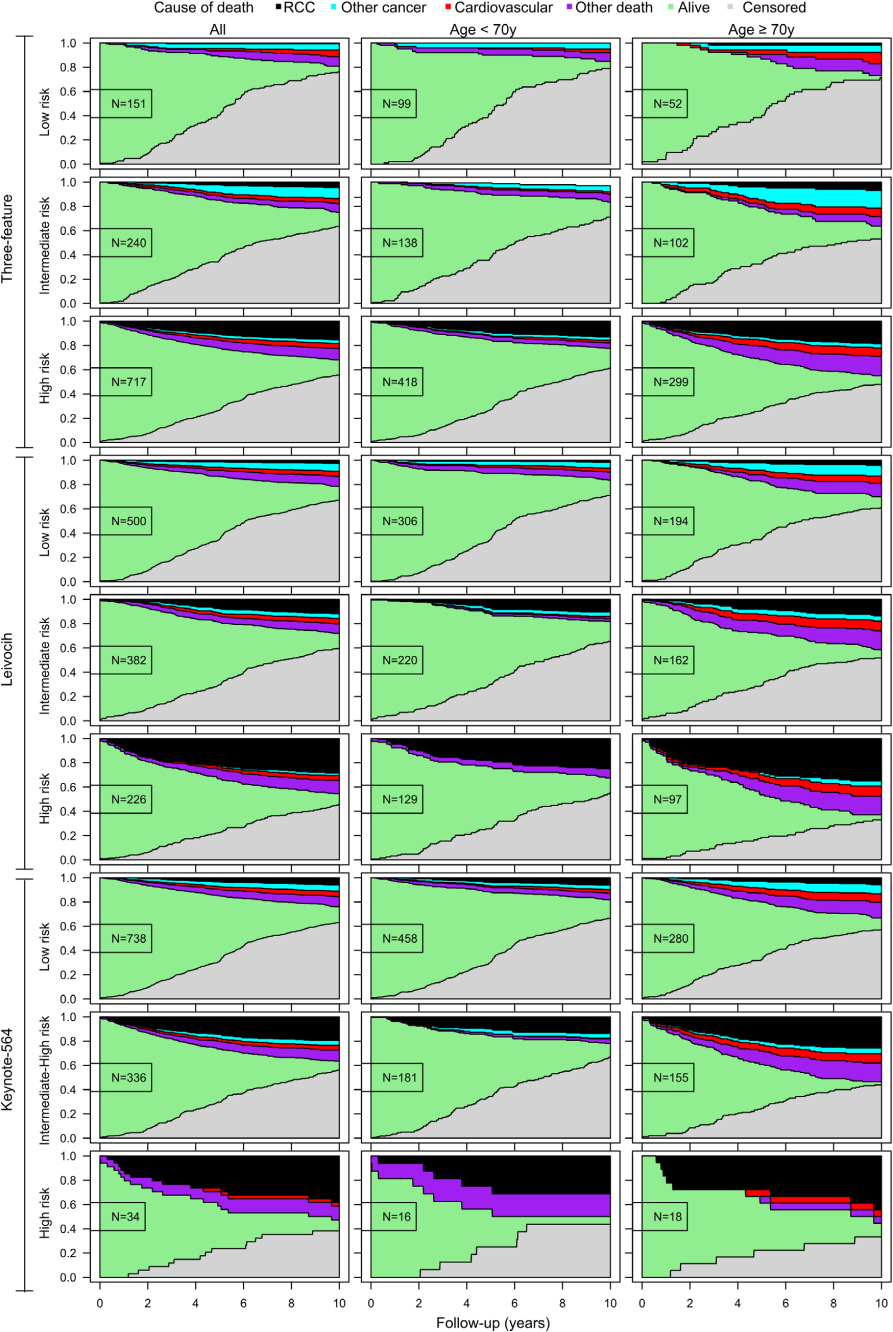
Causes of death during postoperative follow‐up among all patients and patients <70 years and ≥70 years of age at surgery in low, intermediate or intermediate‐high and high‐risk groups according to three‐feature, Leibovich and Keynote‐564 risk models.

**FIGURE 2:**
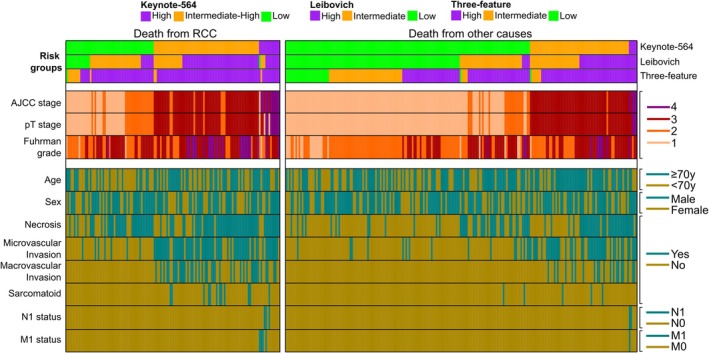
Baseline characteristics and risk groups for disease recurrence in patients who had died from RCC and from other causes.

Continuous variables are presented with median and interquartile range (IQR) or range and categorical variables with number and percentage. Statistical differences were assessed using Fisher's Exact Test (count data) and Kruskal‐Wallis rank test (numeric data) and corresponding p‐values were reported in the tables. Time to death was calculated from surgery to death from RCC or other causes and presented with median and IQR. The Kaplan–Meier method was used to calculate median OS (mOS) estimates with 95% confidence intervals (95%CI) from surgery to all‐cause death (event) or the last follow‐up visit (censored). All analyses were performed with IBM SPSS (version 28) and R statistical software (version 4.4.0). R‐packages CompSurv (version 0.1.0, Laajala T. D. https://github.com/Syksy/CompSurv) and ComplexHeatmap (version 2.20.0; Zuguang Gu. Complex Heatmap Visualization, iMeta, 2022) were used to visualize and process the data for Figures 1 and 2, respectively. Former was used to illustrate the accumulation of end‐point event proportions over follow‐up (RCC death or other death) while accounting for right‐censoring. Latter was used to visualize a heatmap of binary clinical variables with 2 levels or the grading variables with 4 levels, with no further hierarchical clustering or variable transformations. Column sorting and annotations were used to show combinations of increasing risk grades obtained from Keynote‐564, Leibovich and the Three‐feature model, with each column depicting a single patient.

### 2.6 Ethics statement

The study was approved by the Institutional Review Boards of Turku University Hospital (T1402/2023) and Karolinska University Hospital (D‐2024‐02023‐02).

## 3 RESULTS

### 3.1 Baseline characteristics

Baseline characteristics of 1108 patients from Turku and Stockholm are described in Table 1. The median age at surgery was 67 years and 64% were male in the whole study population. Most patients had T1 tumours (T1a 33% and T1b 22%). T3a tumours were also common (28%). 74% of patients had undergone RN and 26% PN. 1% of patients had local lymph node metastases (pN1) and 1% completely resected distant metastases (M1 NED). According to the AJCC staging, 55% had stage I (T1N0M0), 13% had stage II (T2N0M0), 30% had stage III (T1 − 2N1M0 or T3N_any_M0) and 2% had stage IV (T4N_any_M0 or T_any_N_any_M1 NED) tumours. There were significant differences in baseline characteristics and survival outcomes between the Turku and Stockholm cohorts (Table 1).

**TABLE 1:** Baseline characteristics and survival status for the Turku and Stockholm cohorts. For count data, proportions inside a cohort are shown in parenthesis (%), with rounding to the nearest full percentage. For numeric or continuous variables, interquartile range (IQR) is shown in parenthesis. Statistical differences between the two cohorts were assessed using Fisher's exact test (FET) for count data and Kruskal‐Wallis rank test for numeric data.

Variable	Values	Turku cohort	Stockholm cohort	p‐value
Patient count	N	473	635	
Age	Years	69 (60–75)	65 (55–73)	<0.001
Sex	Female	183 (39%)	217 (34%)	0.129
	Male	290 (61%)	418 (66%)	
pT stage	T1a	128 (27%)	240 (38%)	*
	T1b	136 (29%)	112 (18%)	
	T2a	58 (12%)	40 (6%)	
	T2b	25 (5%)	19 (3%)	
	T3a	105 (22%)	201 (32%)	
	T3b‐c	10 (2%)	17 (3%)	
	T4	11 (2%)	6 (1%)	
Tumour diameter	Maximum size (mm)	55 (38–80)	50 (30–75)	0.002
Necrosis	No	290 (61%)	439 (69%)	0.007
	Yes	183 (39%)	196 (31%)	
Fuhrman grade	1	55 (12%)	46 (7%)	0.001
	2	256 (54%)	304 (48%)	
	3	136 (29%)	232 (37%)	
	4	26 (5%)	53 (8%)	
Microvascular invasion	No	372 (79%)	402 (63%)	<0.001
	Yes	101 (21%)	233 (37%)	
Macrovascular invasion	No	416 (88%)	564 (89%)	0.704
	Yes	57 (12%)	71 (11%)	
Sarcomatoid	No	462 (98%)	615 (97%)	0.465
	Yes	11 (2%)	20 (3%)	
pN status	N0/Nx	466 (99%)	632 (100%)	0.108
	N1	7 (1%)	3 (0%)	
M status	M0	469 (99%)	628 (99%)	0.767
	M1 NED	4 (1%)	7 (1%)	
Operation	Radical nephrectomy	411 (87%)	409 (64%)	<0.001
	Partial nephrectomy	62 (13%)	226 (36%)	
OS follow‐up	Days	1524 (609–2874)	1884 (1138–2646)	<0.001
Event type	RCC death	83 (18%)	51 (8%)	<0.001
	Non‐RCC death	97 (21%)	123 (19%)	
	Censored (Alive)	293 (62%)	461 (73%)	

* = FET p‐value could not be calculated due to cells with very low counts.

### 3.2 Causes of death and time to death after surgery

After the median follow‐up of 5.0 years (IQR 2.5–7.6) from surgery, 354 (32%) patients had died; 134 (12%) from RCC, 59 (5%) from other cancers, 54 (5%) from CVD, 75 (10%) from other non‐cancer causes and 32 (3%) from unknown causes. 754 (68%) patients were alive; 646 (58%) without RCC and 108 (10%) with RCC recurrence. A detailed list of other cancers and other non‐cancer causes leading to death is presented in Table S1.

38% of deaths were attributed to RCC, 17% to other cancers, 15% to cardiovascular diseases, 22% to other non‐cancer causes and 9% to unknown causes (Table 2). The median time to RCC death was 3.7 years (IQR 1.6–6.6, range 0.3–14.1). Median times to death from other cancers (3.9 years [IQR 1.9–7.0]), CVD (4.9 years [IQR 2.1–8.3]) and other non‐cancer causes (4.2 years [IQR 1.3–7.7]) were longer (Table 2). A total of 3% of deaths were caused by complications related to surgery with a median time to death of 0.02 years (IQR 0.0–1.7). Deaths from CVD (19%) and other non‐cancer causes (25%) were more common among patients ≥70 years at surgery (Table 2).

**TABLE 2:** Causes of death and time to death after surgery for ccRCC in the whole study population and in different subgroups.

Cause of death	Number of deaths (%)	Time to death after surgery (years)	Deaths Turku	Deaths Stockholm	Deaths <70 years at surgery	Deaths ≥70 years at surgery
	n = 354	Median (IQR)	n = 180	n = 174	n = 150	n = 204
RCC	134 (38%)	3.7 (1.6–6.6)	83 (46%)	51 (29%)	66 (44%)	68 (33%)
Other cancer	59 (17%)	3.9 (1.9–7.0)	22 (12%)	37 (21%)	29 (19%)	30 (15%)
Cardiovascular disease	54 (15%)	4.9 (2.1–8.3)	22 (12%)	32 (18%)	16 (11%)	38 (19%)
Other non‐cancer	75 (22%)	4.2 (1.3–7.7)	40 (22%)	35 (20%)	24 (16%)	51 (25%)
Unknown	32 (9%)	4.2 (2.0–7.4)	13 (7%)	19 (11%)	15 (10%)	17 (8%)

The estimated mOS after radical surgery was 10.2 years (95%CI 9.7–10.8) for all patients. 72.8%, 64.5%, 62.6% and 44.0% of patients with stage I, II, III and IV RCC at surgery were alive at the end of follow‐up. A total of 5.5%, 20.6%, 18.5% and 40.0% of patients with stage I, II, III and IV RCC at surgery had died from RCC and 21.7%, 14.9%, 18.8% and 16.0% had died from non‐RCC causes, respectively. The mOS was 10.7 years (95%CI 10.2–11.3) for stage I, 10.1 years (95%CI 8.6–11.6) for stage II, 9.0 years (95%CI 7.6–10.5) for stage III and 5.1 years (95%CI 0–10.1) for patients with stage IV RCC. Among 11 patients with M1NED, the mOS was 9.7 years (95%C 5.0–14.4). Among 256 patients with RCC recurrence, the mOS was 6.8 years (95%CI 5.3–8.3) from surgery and 3.5 years (95%CI 2.3–4.6) from RCC recurrence.

### 3.3 Causes of death in different risk groups for RCC recurrence

67% of patients were classified as having a low risk of recurrence according to Keynote‐564 criteria compared to 14% and 45% according to Three‐feature and Leibovich models (Table 3). 41% of RCC deaths were observed in the low‐risk group according to Keynote‐564 criteria as opposed to 1% and 12% in the low‐risk groups of three‐feature and Leibovich models (Table 3). During postoperative follow‐up, there were deaths from non‐RCC causes among all risk groups (Table 3, Figure 1), and this was more pronounced in patients ≥70 years at surgery (Figure 1).

**TABLE 3:** The rate of deaths due to RCC or other causes and the median time to death stratified according to the risk classifiers.

Risk model	Risk group	Number of patients (%)	Number of observed deaths from RCC (%)	Time to death from RCC (years)	Number of observed deaths from other causes (%)	Time to death from other causes (years)
			n = 134	Median (IQR)	n = 220	Median (IQR)
Three‐feature	Low	151 (14%)	1 (1%)	2.8 (2.8–2.8)	29 (13%)	4.6 (1.7–7.3)
	Intermediate	240 (22%)	12 (9%)	5.2 (3.4–7.6)	56 (25%)	4.5 (2.5–8.3)
	High	717 (65%)	121 (90%)	3.6 (1.6–6.3)	135 (61%)	3.7 (1.7–7.6)
Leibovich	Low	500 (45%)	16 (12%)	5.8 (2.8–9.3)	109 (50%)	4.5 (2.0–7.9)
	Intermediate	382 (34%)	53 (40%)	4.7 (3.0–7.8)	70 (32%)	3.2 (1.6–7.4)
	High	226 (20%)	65 (49%)	1.9 (1.0–4.9)	41 (19%)	4.2 (2.0–6.8)
Keynote‐564	Low	738 (67%)	55 (41%)	5.2 (3.1–9.4)	153 (70%)	4.5 (1.9–8.1)
	Intermediate‐high	336 (30%)	66 (49%)	2.4 (1.1–5.1)	62 (28%)	3.8 (2.0–7.0)
	High	34 (3%)	13 (10%)	2.2 (0.9–5.1)	5 (2%)	1.8 (0.0–4.3)

### 3.4 Risk factors for death from RCC

Baseline characteristics and risk groups for disease recurrence in patients who had died from RCC and from other causes were illustrated in Figure 2. Younger age at surgery (p = 0.046) and tumours with higher AJCC and pT stage (p < 0.001), higher Fuhrman grade (p < 0.001), necrosis (p < 0.001), microvascular invasion (p < 0.001), macrovascular invasion (p < 0.001) and sarcomatoid differentiation (p = 0.008) were significantly more common in patients with death from RCC (Figure 2, Table S2).

## 4 DISCUSSION

After the median postoperative follow‐up of five years, the estimated median OS was 10.2 years, all‐cause mortality was 32% and RCC mortality 12% in a population of 1108 patients surgically treated with curative intent for ccRCC in Finland and Sweden. RCC was the most common cause of death (38%), but the majority of deaths were attributed to non‐RCC causes, most commonly other cancers (17%) and cardiovascular diseases (15%), as observed earlier in localized RCC.^14^, ^18^ The median time to RCC death was 3.7 years, but 25% of RCC deaths were observed after six years from surgery consistent with a 20% incidence of late recurrences.^19^ We observed that deaths from CVD and other non‐cancer causes happened later (median time 4.9 and 4.2 years and 25% after 8.3 and 7.7. years from surgery, respectively) as also seen in the US.^14^ CVD and other non‐cancer deaths (19% and 25% of all deaths) were frequently seen in patients ≥70 years at surgery but also in patients <70 years at surgery (11% and 16% of all deaths).

When patients were stratified according to the risk of recurrence, we observed that the group of patients classified as having a low risk of recurrence according to Keynote‐564 criteria (67%) was large compared to Three‐feature (14%) and Leibovich (45%) models. To our knowledge, this was the first study comparing stratification criteria used in the Keynote‐564 study with other prognostic models. Adjuvant therapy was not used outside clinical trials in the Nordic countries during this study. A total of 23% of patients developed disease recurrence with the mOS of only 3.5 years from RCC recurrence despite TKIs being available since 2006 and immunotherapies since 2015 for the treatment of mRCC in Europe. Surprisingly, patients with M1 NED had long survival (mOS 9.7 years) in this study supporting surgery in this highly selected, small subgroup.

After improved OS yielded with adjuvant pembrolizumab in the Keynote‐564 trial, adjuvant therapy was adopted into clinical practice in the Nordic countries according to international treatment guidelines.^11^, ^20^ Patients who fulfil the inclusion criteria of the Keynote‐564 trial are considered for adjuvant therapy in multidisciplinary meetings involving urologists, oncologists, radiologists and pathologists. The individual risk of disease recurrence is assessed using validated prognostic nomograms (e.g., Leibovich model^5^), and the benefit of adjuvant therapy is weighed against the risk of immune‐related adverse events with each patient. Despite adjuvant therapy, some patients still develop disease progression (estimated four‐year disease‐free survival 64.9% in pembrolizumab arm vs. 56.6% in placebo arm^12^). Molecular profiling has revealed prognostic information but is not yet adopted into clinical practice in RCC.^21^ Hopefully, biomarkers will improve patient selection for perioperative immunotherapy in RCC as seen in urothelial and lung cancers.^22^, ^23^ Notably, 41% of RCC deaths in this study population occurred in patients excluded from adjuvant therapy according to Keynote‐564 criteria highlighting the need for more accurate risk prediction. Leibovich and Three‐feature models could possibly define the population with a higher risk of death from RCC as 88–90% of deaths from RCC occurred in Leibovich intermediate or high‐risk and Three‐feature high‐risk groups, but this finding needs to be validated in other patient cohorts.

In addition to adjuvant therapy, it would be worthwhile to pay attention to comorbidities and risk factors such as smoking, hypertension and physical inactivity after surgery for RCC. Smoking was associated with shorter OS after radical or cytoreductive nephrectomy, and quitting smoking after RCC diagnosis reduced RCC mortality.^24^, ^25^ It is also reported that 5% of patients developed hypertension and 4% experienced major cardiovascular events within five years from nephrectomy^26^ consistent with the 5% incidence of CVD deaths in this study. Multidisciplinary interventions involving specialized nurses, physiotherapists and general practitioners aiming to optimize the treatment of comorbidities and lifestyle factors could improve OS even further regardless of the risk of RCC recurrence after nephrectomy.

The limitations of this study are attributed to its retrospective design. Performance status and the Charlson comorbidity index^27^ at the time of surgery and information about systemic treatments for mRCC during postoperative follow‐up were omitted from our analyses. Although other cancers were usually histologically verified, there might be some RCC deaths incorrectly classified as deaths from other cancers, e.g., from cholangiocarcinoma and pancreatic cancer. Despite differences in baseline characteristics, RCC mortality increased similarly according to the risk of recurrence and non‐RCC deaths were observed among all risk groups in both cohorts consistent with findings reported from the Recur database.^28^ Moreover, the 23% rate of recurrences in this study was similar to 22.6% in the Recur data.^28^ Therefore, we think that the findings of this study could be generalized into similar patient populations.

## 5 CONCLUSIONS

All‐cause mortality was 32% and RCC mortality 12% after the median follow‐up of five years from surgery with curative intent for ccRCC. 41% of deaths from RCC were observed in patients currently excluded from adjuvant therapy suggesting that more research on patient selection for perioperative immunotherapy is needed. As non‐RCC deaths were observed among all risk groups, multidisciplinary interventions improving the treatment of comorbidities and lifestyle factors after nephrectomy could prolong OS even more.

## AUTHOR CONTRIBUTIONS

Conception and design: KEM, PJ, PL, AK, ML. Acquisition of data: AB, PL, AK, IM, PV, KEM. Analysis and interpretation of data: AB, IM, TDL, KEM. Drafting of the manuscript: AB, TDL, KEM. Critical revision of the manuscript for important intellectual content: IM, PV, ML, AK, PL, PJ. Statistical analysis: TDL, KEM. Obtaining funding: KEM. Administrative, technical or material support: None. Supervision: KEM, PJ, PL, AK, ML. Other: None.

## CONFLICT OF INTEREST STATEMENT

KEM has received consulting or advisory honoraria from Astellas, Bayer, Bristol‐Myers Squibb, GlaxoSmithKline, Ipsen, Janssen, Merck Sharp & Dohme, Merck−Pfizer alliance, Novartis, Roche and Sanofi. Other authors have no conflicts of interest to declare.

## DATA SHARING

The data that support the findings of this study are available from the corresponding author upon reasonable request.

## Supporting information


**Table S1.** Deaths from other cancer and other non‐cancer causes

**Table S2.** Key variables stratified according to the cause of death. Reported p‐values are Fisher's Exact Test for counts between non‐RCC and RCC‐specific death and variable levels.
