# Supplementary material for: Competing risk of death in patients with low, intermediate and high risk of recurrence after radical surgery for clear cell renal cell carcinoma
Source: BJUI Compass. 2025 Jul 21;6(7):e70047. doi: 10.1002/bco2.70047 (PMC12279469; doi:10.1002/bco2.70047)
Supplement: Supplementary file 1 — Table S1. Deaths from other cancer and other non‐cancer causes [file BCO2-6-e70047-s001.docx]

Supplementary Table 1. Deaths from other cancer and other non-cancer causes

| Deaths from other cancer | Number of cases | % of other cancer deaths |
| --- | --- | --- |
| Acute myeloid leukemia | 1 | 1.7% |
| Angiosarcoma | 1 | 1.7% |
| Breast cancer | 3 | 5.1% |
| Cholangiocarcinoma | 10 | 16.9% |
| Colorectal cancer | 4 | 6.8% |
| Esophageal cancer | 2 | 3.4% |
| Gastric cancer | 4 | 6.8% |
| Hepatocellular carcinoma | 1 | 1.7% |
| Lung cancer | 7 | 11.9% |
| Melanoma | 4 | 6.8% |
| Mesothelioma | 3 | 5.1% |
| Myeloma | 2 | 3.4% |
| Neuroendocrine tumor | 1 | 1.7% |
| Non-Hodgkin lymphoma | 1 | 1.7% |
| Ovarian cancer | 2 | 3.4% |
| Pancreatic cancer | 7 | 11.9% |
| Prostate cancer | 4 | 6.8% |
| Unknown primary | 1 | 1.7% |
| Urothelial cancer | 1 | 1.7% |

| Deaths from other non-cancer causes | Number of cases | % of non-cancer deaths |
| --- | --- | --- |
| COPD | 4 | 5.3% |
| Dementia | 13 | 17.3% |
| Gastrointestinal disease | 3 | 4% |
| Infection | 29 | 38.7% |
| Kidney failure | 2 | 2.7% |
| Liver disease | 3 | 4.0% |
| Lung fibrosis | 1 | 1.3% |
| Neurological disease | 2 | 2.7% |
| Postoperative complications | 12 | 16.0% |
| Traumatic | 6 | 8.0% |
