# Supplementary material for: Competing risk of death in patients with low, intermediate and high risk of recurrence after radical surgery for clear cell renal cell carcinoma
Source: BJUI Compass. 2025 Jul 21;6(7):e70047. doi: 10.1002/bco2.70047 (PMC12279469; doi:10.1002/bco2.70047)
Supplement: Supplementary file 2 — Table S2. Key variables stratified according to the cause of death. Reported p‐values are Fisher's Exact Test for counts between non‐RCC and RCC‐specific death and variable levels. [file BCO2-6-e70047-s002.docx]

**Supplementary Table 2**. Key variables stratified according to the cause of death. Reported p-values are Fisher’s Exact Test for counts between non-RCC and RCC-specific death and variable levels.

| **Variable** | **Values** | **All patients with observed death** | **Non-RCC death** | **RCC death** | **p-value** |
| --- | --- | --- | --- | --- | --- |
| N |  | 354 | 220 | 134 |  |
| Age (years) | below 70 | 150 (42%) | 84 (38%) | 66 (49%) | 0.046 |
|  | 70 or above | 204 (58%) | 136 (62%) | 68 (51%) |  |
| AJCC stage | 1 | 167 (47%) | 133 (60%) | 34 (25%) | <0.001 |
|  | 2 | 50 (14%) | 21 (10%) | 29 (22%) |  |
|  | 3 | 123 (35%) | 62 (28%) | 61 (46%) |  |
|  | 4 | 14 (4%) | 4 (2%) | 10 (7%) |  |
| pT stage | 1 | 169 (48%) | 133 (60%) | 36 (27%) | <0.001 |
|  | 2 | 50 (14%) | 21 (10%) | 29 (22%) |  |
|  | 3 | 124 (35%) | 63 (29%) | 61 (46%) |  |
|  | 4 | 11 (3%) | 3 (1%) | 8 (6%) |  |
| Fuhrman grade | 1 | 26 (7%) | 22 (10%) | 4 (3%) | <0.001 |
|  | 2 | 151 (43%) | 110 (50%) | 41 (31%) |  |
|  | 3 | 143 (40%) | 78 (35%) | 65 (49%) |  |
|  | 4 | 34 (10%) | 10 (5%) | 24 (18%) |  |
| Sex | Female | 133 (38%) | 81 (37%) | 52 (39%) | 0.735 |
|  | Male | 221 (62%) | 139 (63%) | 82 (61%) |  |
| Necrosis | No | 186 (53%) | 142 (65%) | 44 (33%) | <0.001 |
|  | Yes | 168 (47%) | 78 (35%) | 90 (67%) |  |
| Microvascular invasion | No | 226 (64%) | 161 (73%) | 65 (49%) | <0.001 |
|  | Yes | 128 (36%) | 59 (27%) | 69 (51%) |  |
| Macrovascular invasion | No | 295 (83%) | 200 (91%) | 95 (71%) | <0.001 |
|  | Yes | 59 (17%) | 20 (9%) | 39 (29%) |  |
| Sarcomatoid differentiation | No | 337 (95%) | 215 (98%) | 131 (92%) | 0.008 |
|  | Yes | 17 (5%) | 5 (2%) | 12 (8%) |  |
| N1 status | N0 | 349 (99%) | 218 (99%) | 131 (98%) | 0.371 |
|  | N1 | 5 (1%) | 2 (1%) | 3 (2%) |  |
| M1 status | M0 | 359 (99%) | 219 (100%) | 130 (97%) | 0.070 |
|  | M1 | 5 (1%) | 1 (0%) | 4 (3%) |  |
